# Effect of Dietary Macronutrients on Postprandial Glucagon and Insulin Release in Obese and Normal-Weight Women

**DOI:** 10.1155/2020/4603682

**Published:** 2020-04-30

**Authors:** Tomasz Wikarek, Piotr Kocełak, Aleksander J. Owczarek, Jerzy Chudek, Magdalena Olszanecka-Glinianowicz

**Affiliations:** ^1^Health Promotion and Obesity Management Unit, Department of Pathophysiology, Medical Faculty in Katowice, The Medical University of Silesia, Katowice, Poland; ^2^Department of Gynecology and Obstetrics, Medical Faculty in Katowice, The Medical University of Silesia, Katowice, Poland; ^3^Department of Statistics, Faculty of Pharmaceutical Sciences in Sosnowiec, Medical University of Silesia, Katowice, Poland; ^4^Pathophysiology Unit, Department of Pathophysiology, Medical Faculty in Katowice, The Medical University of Silesia, Katowice, Poland; ^5^Department of Internal Medicine and Oncological Chemotherapy, Medical Faculty in Katowice, The Medical University of Silesia, Katowice, Poland

## Abstract

The aim of the study was to assess the effect of dietary macronutrients on circulating glucagon and insulin levels in obese and normal-weight women. Potentially, the impaired release of glucagon may proceed abnormal glucose metabolism in obese patients ahead of overt diabetes. In 20 insulin-sensitive women (11 obese and 9 normal-weight), plasma concentrations of insulin and glucagon levels were assessed before and after 3 different macronutrient test meals. AUC_total_ insulin in the obese group was increased after protein and carbohydrates compared to fatty test meal consumption (3981 ± 2171 and 4869 ± 2784 vs. 2349 ± 1004 *μ*IU*∗*h/m, *p* < 0.05, respectively), but without a difference between protein and carbohydrates ingestion. However, in the normal-weight group, AUC_total_ insulin was increased after carbohydrates compared to fatty test meal ingestion (3929 ± 1719 vs. 2231 ± 509 *μ*IU*∗*h/ml, *p* < 0.05) and similar after carbohydrate and protein as well as after fatty and protein test meals (3929 ± 1719 vs. 2231 ± 509 vs. 3046 ± 1406 *μ*IU*∗*h/ml, respectively). However, AUC_total_ insulin was significantly increased in obese compared to normal-weight women only after carbohydrate test meal ingestion (4869 ± 2784 vs. 3929 ± 1719 *μ*IU*∗*h/ml, *p* < 0.05). AUC_total_ glucagon was similar after carbohydrate, protein, and fatty test meals ingestion in obese and normal-weight women (921 ± 356 vs. 957 ± 368 vs. 926 ± 262 ng*∗*h/ml and 1196 ± 14 vs. 1360 ± 662 vs. 1792 ± 1176 ng*∗*h/ml, respectively). AUC_total_ glucagon was significantly lower in obese than normal-weight women after a fatty meal (926 ± 262 vs. 1792 ± 1176 ng*∗*h/ml, *p* < 0.01). Postprandial glucagon secretion is not related to the macronutrient composition of the meal in normal-weight women since postprandial glucagon concentrations were stable and did not change after carbohydrate, protein, and fatty test meals. Lower glucagon secretion was observed in obese subjects after fatty meal consumption when compared to normal-weight subjects. Postprandial insulin profile was significantly higher after carbohydrate than fatty test meal intake in the obese group and did not differ between obese and normal-weight groups after carbohydrate, protein, and fatty test meals consumption. Impaired glucagon secretion after fatty meat suggests early pancreatic alpha-cell dysfunction, after a carbohydrate meal is a compensatory mechanism.

## 1. Introduction

Glucagon is a 29-amino-acid peptide released by the pancreatic *α* cells with an antagonistic action to insulin, which has a hyperglycemic effect by enhancement of gluconeogenesis and glycogenolysis in the liver [1].

Physiologically, the serum concentration of glucagon is the highest in the morning, in a fasting state, and decreases postprandially. The stimulants for glucagon release are amino acids, catecholamines, corticosteroids, and intestinal hormones including cholecystokinin, gastrin, and GIP, as well as adrenergic activation in hypoglycemia, whereas glucose and free fatty acids inhibit its release [1].

Insulin, an opponent pancreatic hormone, participating in the regulation of glucose homeostasis, is mainly secreted by the beta cells stimulated by incretin hormones (GLP-1—glucagon‐like peptide‐1 and GIP—glucose‐dependent insulinotropic polypeptide) released by enteroendocrine cells of the gut in response to nutrient absorption [2, 3]. The phenomenon of greater stimulation of insulin release after an oral glucose load than intravenous glucose infusion is called the incretin effect [2].

Meal volume and its composition, including the composition of amino acids, determine glucagon release postprandially. The main place of glucagon action is the liver. However, its receptors have been also identified in the pancreatic *β* cells, heart, kidneys, brain, intestine, adrenal glands, vessels, and adipose tissue [4].

Interestingly, incretin hormones modulate also glucagon secretion. GLP-1 suppresses its secretion, especially in a hyperglycemic state [5], whereas GIP was found to stimulate glucagon secretion [6] to more extent with lower glucose concentration. Moreover, intravenous glucose infusion suppresses glucagon secretion more than oral glucose load at least in healthy subjects [7, 8].

One of the important glucagon actions is the regulation of body mass homeostasis by the impact on satiety sensation and consumed meal size, shown in both rats and humans [1, 9–11]. It seems that glucagon exerts its action on satiety centrally by inhibiting ghrelin action, depending on the consumed meal size [12, 13]. Ghrelin stimulates neuropeptide Y and agouti-related protein release and in consequence decreases satiety and increases hunger [14–16]. It is suggested that impaired effect of glucagon on satiety may be important in diabetic and obese subjects, contributing to the further increase of body fat accumulation due to the altered sensation of postprandial satiety and enhanced food intake. However, more recent data showed that glucagon-induced satiety is preserved in type 1 diabetic patients but lowered in obese regardless of insulin release [13]. The role of glucagon in the development of diabetes is widely accepted and supported by many studies that showed its role in the regulation of body mass and energy expenditure by its central action on food intake. Moreover, some new data showed a potential role of glucagon receptor antagonists in the management of obesity and diabetes [17, 18]. Higher fasting plasma glucagon levels and the lack of its postprandial suppression or even enhanced secretion were shown in subjects with type 2 diabetes [19]. One study found differences in glucagon release in diabetic obese and normal-weight subjects after a mixed test meal intake. Higher fasting glucagon concentration and postprandial glucagon release were observed in obese than in nonobese diabetic subjects. Besides, a significant positive correlation between fasting glucagon levels and BMI in normal-weight and obese diabetic subjects was shown [20].

Previous studies have reported diminished incretin effect in obesity even in the lack of abnormal glucose metabolism [21] due to a reduced release of GLP-1 after nutrient ingestion [22] and reduced responsiveness to GIP, since higher postprandial GIP concentration was detected in obesity which potentially may stimulate hunger [23].

In our previous study, we analyzed postprandial glucagon-like peptide-1 (GLP-1) and glucose-dependent insulinotropic polypeptide (GIP), but not glucagon release [24] after ingestion of different types of meals as potentially different macronutrients may attenuate not only insulin release but also a sensation of satiety and hunger.

The abnormal release of gut hormones is observed in type 2 diabetes; however, this abnormality may proceed with the development of diabetes and be the first sign of the development of hyperglycemia. It seems that abnormal glucagon release may also occur in obese subjects ahead of the development of type 2 diabetes.

Therefore, the present study aimed to assess the effect of dietary macronutrients on postprandial glucagon and insulin release in obese and normal-weight nondiabetic young women.

## 2. Materials and Methods

### 2.1. Experimental Methods

The study included eleven obese and nine healthy normal-weight subjects as described in detail previously [24]. The protocol of inclusion and exclusion criteria was similar to the protocol presented in the previous paper [24].

The characteristics of the study groups are presented in Table 1.

### 2.2. Study Protocol

All the subjects were served carbohydrate, protein, and fatty test meals. The procedure and content of the test meals were described previously [24].

All the procedures were approved by the Bioethical Committee. All the study participants gave their informed consent before enrollment into the study.

### 2.3. Laboratory Procedures

Venous blood samples were collected for biochemical measurements in the manner described previously [24]. Besides, plasma glucagon was assessed by EIA (Phoenix Pharmaceuticals, Inc., US) with a lower limit of sensitivity of 0.28 ng/ml, respectively, and intra- and interassay CV of 5.0–10.0 and <15%, respectively.

### 2.4. Statistical Analysis

All statistical analyses were previously described in detail [24]. The *p* value less than 0.05 was set as statistically significant. The power (*β*) of all tests are assured to be not less than 70%.

## 3. Results

### 3.1. Changes in Plasma Glucagon Concentrations after Test Meals

Fasting plasma glucagon levels did not differ before ingestion of all the test meals and the consumption of the meals did not change its concentration in both study groups (Table 1).

**Figure 1:**
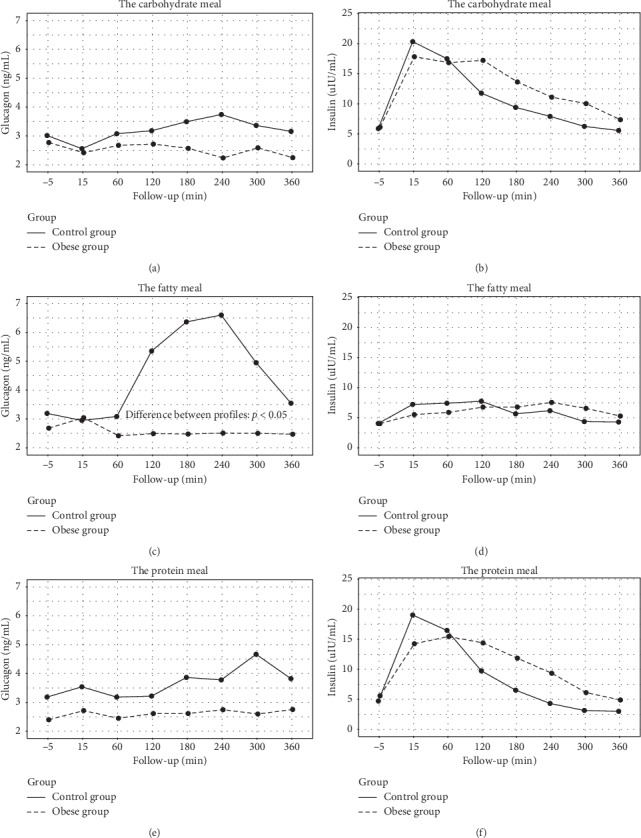
Plasma glucagon concentrations before and during a 6 h period after consumption of the test meals. Carbohydrate meal: (a) glucagon and (b) insulin levels. Fatty meal: (c) glucagon and (d) insulin levels. Protein meal: (e) glucagon and (f) insulin levels.

Postprandial glucagon concentrations did not change after ingestion of carbohydrate and protein test meals when compared to fasting concentrations in the study and control groups. There were also no differences in their concentrations after carbohydrate and protein meals between study and control groups, while postprandial glucagon levels were significantly higher after fatty meal ingestion in normal-weight than in the obese group (*p* < 0.05) (Figure 1).

There were no differences in postprandial AUC plasma glucagon concentrations between carbohydrate and protein in relation to a fatty meal and after carbohydrate and protein meals consumption in both the study and control groups. Only after fatty meal consumption, postprandial AUC glucagon concentration was significantly higher in normal-weight than in the obese group (*p* < 0.01) (Table 2; Figure 1).

The mean time to peak glucagon concentration after fatty test meal was 172 ± 48 minutes in the normal-weight group. However, it was not possible to determine peak glucagon concentration in the obese group due to a flat postprandial profile.

There was no correlation between peak plasma glucagon concentration and maximum satiety sensation as well as minimum hunger sensation after fatty meal consumption in the control group.

The estimated return time of plasma glucagon to fasting concentration based on the approximation function (*y*=*A*_1_*∗t*^4^+*A*_2_*∗t*^3^+*A*_3_*∗t*^2^+*A*_4_*∗t*+*A*_5_), where *A*_*i*_ is a value of glucagon/insulin in the *i*-th time point of measurement and *t* is time, and it was 6 h in the control group and 9 h 52 min in the study group. The goodness of fit was very good with *R*^2^ = 0.994.

### 3.2. Changes in Plasma Insulin Concentrations after Test Meals

There were no differences in fasting insulin concentrations before test meals administration within the obese and control groups and between groups. However, its level was significantly higher in the obese group, but not in the normal-weight group, before consumption of the carbohydrate than the fatty test meal intake (Table 1).

Postprandial insulin profile did not differ between both groups after all test meals consumption. However, postprandial insulin profile was significantly higher after carbohydrate than fatty test meal intake, but only in the obese group (*p* < 0.01) (Figure 1).

The AUC of total insulin value was higher after the protein and carbohydrate than the fatty test meal intake (*p* < 0.05) in the obese group and did not differ after ingestion of the protein and carbohydrate test meals, while in the normal-weight group, the AUC of total insulin value was higher only after the carbohydrate than the fatty test meal intake (*p* < 0.05) and did not differ after the carbohydrate and protein test meals as well as the protein and fatty test meals intake. The AUC of total insulin value was significantly higher in obese women than in normal-weight women only after the carbohydrate test meal intake (*p* < 0.05) (Table 2).

Time to maximum postprandial serum insulin concentration was similar after carbohydrate and protein test meals intake in obese and normal-weight groups (61 ± 47 *vs*. 34 ± 16 min and 46 ± 19 *vs*. 32 ± 16 min, respectively).

Based on a linear regression model analysis, there were no differences in the estimated time to return to fasting insulin concentrations after protein and carbohydrate test meals intake in both groups. There were also no differences in estimated time between the obese and normal-weight groups after carbohydrate and protein test meals intake (10 h and 10 min *vs*. 7 h and 24 min; 8 h and 43 min vs. 6 h and 4 min, respectively).

There were no correlations between anthropometric parameters and log_10_ of fasting and AUC of glucagon concentrations after carbohydrate and protein test meals intake. The log_10_ of postprandial glucagon concentrations was inversely proportional to BMI and fat mass (*r* = −0.60, *p* < 0.01, and *r* = −0.57, *p* < 0.01, respectively, and *r* = −0.62, *p* < 0.01, and *r* = −0.60, *p* < 0.01, respectively) after fatty test meal intake.

In the obese group, there were positive correlations between AUC of postprandial glucagon concentration and plasma insulin concentrations (*r* = 0.62, *p* < 0.05, respectively) after a fatty meal.

## 4. Discussion

In our previous study [24]) the protocol of the study was similar as in the present study. Three different solid meals were given to all the subjects, which consisted of 93% energy of carbohydrate, 72% energy of protein, and 84% energy of fat in a sequence parallel with visual scoring of satiety sensation. The results of the study [24] showed that the reduced GLP-1 release after consumption of a fatty meal in obese women may explain the impaired sensation of satiety, and the impaired postprandial GIP release may be the early indicator of incretin axis dysfunction in obese women.

Our study shows similar fasting glucagon levels in obese and normal-weight women, but flat glucagon postprandial profile in the obese group, and significantly lower postprandial glucagon levels after fatty test meal intake in obese may represent an adaptive mechanism preventing an increase in postprandial glucose concentration in the early stage of insulin resistance without compensative hyperinsulinemia.

Fasting plasma glucagon levels were similar before ingestion of all the test meals in both study groups and did not differ between the obese and normal-weight women. In addition, we observed no postprandial differences in glucagon levels, as well as its postprandial AUC after carbohydrate and protein meals intake between the obese and normal-weight groups, and only after fatty meal intake glucagon levels and its postprandial AUC were lower in obese than normal-weight women. Moreover, our results showed that log_10_ of AUC of postprandial glucagon concentration after fatty meal intake was inversely proportional to BMI and fat mass. It should be noted that a previously published study showed divergent results. In this study, postprandial glucagon levels were higher after fatty meal consumption in obese than normal-weight women [25]. However, our previously published study showed that postprandial incretin axis dysregulation precedes insulin resistance and hyperinsulinemia development in obese. It suggests that lower postprandial GIP levels may prevent enhanced glucagon secretion in the early stage of insulin resistance [24]. This hypothesis is confirmed by the results of studies that showed that GIP is the main stimulus for glucagon release in type 2 diabetic subjects [26]. Moreover, physiologically GLP-1 is a glucagon-release inhibitor [27].

The return time of plasma glucagon to fasting concentration could only be estimated in normal-weight subjects after high-fat meal consumption, whereas in obese, it was not possible to estimate the time as a flat course of postprandial glucagon concentration was observed irrespective of meal composition. It may be explained partially as the mechanism delaying the occurrence of postprandial hyperglycemia in insulin-resistant subjects with subsequent hyperinsulinemia. In a study on the influence of liquid high-protein meal (510 kcal, of which 180 from soy protein), the peak time for glucagon concentration was 35 minutes regardless of BMI and age. In that study, it was shown that liquid protein meal was stimulating glucagon secretion proportionally to its protein content but independently from BMI [28]. No differences were observed in postprandial AUC of plasma glucagon concentrations after test meals consumption in both study and control groups, irrespective of BMI and higher fasting and postprandial glucagon concentrations observed in men; however, the influence of sex on glucagon is not well established as some investigators showed higher insulin-induced concentrations in men with comparable fasting levels suggesting enhanced counterregulatory effects [28–31], whereas others found no differences in fasting and exercise [32] or hypoglycaemia-induced glucagon concentrations [33]. As mentioned above, in our study, there were no differences in AUC of postprandial glucagon concentrations after test meals in both study groups. The divergent results may stem from a small study cohort and their heterogeneity with the presence of insulin resistance in studied subjects, as well as mixed macronutrient content of test meals.

It is worth to mention that fatty meal was the greatest stimulator of glucagon secretion in normal-weight subjects. An interesting finding shown in our study is that the log_10_ of postprandial glucagon concentrations was inversely proportional to BMI and fat mass after fatty test meal intake. In contrast, in a study performed in diabetic subjects, a positive correlation between fasting glucagon concentration and BMI and higher fasting glucagon level and postprandial AUC in obese than nonobese diabetics were showed [20].

Even though no differences were shown for AUC postprandial insulin concentrations, the result of previously published study [34] showed that high carbohydrate meal stimulates an early phase of glucagon secretion and high-fat meals induce a late phase of glucagon release. It seems that lower postprandial glucagon concentration in obese women after a fat meal in our study may be an early sign of pancreatic alpha-cell dysfunction, or it may represent a counterregulatory mechanism to the development of insulin resistance. This hypothesis is supported by positive correlations between the AUC of postprandial glucagon concentration and plasma insulin concentrations after a fatty meal in the obese group.

An interesting result obtained in our study is longer estimated return time of plasma glucagon to fasting concentration in obese than the normal-weight group. It suggests the next step of disturbances in postprandial glucagon release. However, further studies are necessary to confirm this observation.

### 4.1. Limitation of the Study

The limitation of this study is the small sample size and only female participants enrolled for better heterogeneity. Moreover, test meals energy content and density were divergent and we did not evaluate with high-fibre test meal. We did not measure daily energy consumption and content throughout the examination as well as the kinetics of gastric emptying. Furthermore, test meals were not administered randomly.

## 5. Conclusion

Postprandial glucagon secretion is not related to the macronutrient composition of the meal in normal-weight women since postprandial glucagon concentrations were stable and did not change after carbohydrate, protein, and fatty test meals.

Lower glucagon secretion was observed in obese subjects after fatty meal consumption when compared to normal-weight subjects.

Postprandial insulin profile was significantly higher after carbohydrate than fatty test meal intake in the obese group.

Postprandial insulin secretion did not differ between obese and normal-weight groups after carbohydrate, protein, and fatty test meals consumption.

Impaired glucagon secretion after fatty meat suggests early pancreatic alpha-cell dysfunction, while higher insulin secretion after a carbohydrate meal is a compensatory mechanism for developing insulin resistance in obese subjects.

## Figures and Tables

**Table 1:** Characteristics of the study groups (mean values and standard deviations).

	Obese (*N* = 11)	Normal-weight (*N* = 9)	
	Mean ± SD	Mean ± SD	*p* value
Age (years)	28.9 ± 5.7	23.3 ± 5.3	<0.05
Body mass (kg)	90.9 ± 11.5	59.1 ± 7.3	<0.001
BMI (kg/m^2^)	33.3 ± 4.4	22.6 ± 2.0	<0.001
Body fat (kg)	44.9 ± 5.3	29.2 ± 4.0	<0.001
Body fat (%)	40.6 ± 8.5	17.3 ± 3.0	<0.001
Waist circumference (cm)	104.0 ± 8.2	74.8 ± 2.2	<0.001

*Fasting serum glucose (mg/L)*			
Before carbohydrate meal administration	841 ± 75	848 ± 98	NS
Before protein meal administration	844 ± 62	799 ± 98	NS
Before fatty meal administration	838 ± 66	836 ± 63	NS

*Fasting serum insulin (μIU/mL)*			
Before carbohydrate meal administration	6.1 ± 2.4	5.9 ± 2.7	NS
Before protein meal administration	5.6 ± 1.9	4.7 ± 3.6	NS
Before fatty meal administration	4.0 ± 1.5	4.1 ± 1.6	NS

*Fasting plasma glucagon (ng/mL)*			
Before carbohydrate meal administration	3.01 ± 1.99	2.77 ± 0.75	NS
Before protein meal administration	3.18 ± 1.74	2.40 ± 0.37	NS
Before fatty meal administration	3.19 ± 1.49	2.68 ± 0.86	NS

**Table 2:** Effect of the test meals on insulin and glucagon release (AUC value) in obese (*N* = 11) and normal-weight (*N* = 9) subjects (mean values and standard deviations).

	Row data			Energy-adjusted data		
	Carbohydrate	Protein	Fatty	Carbohydrate	Protein	Fatty
	Mean ± SD	Mean ± SD	Mean ± SD	Mean ± SD	Mean ± SD	Mean ± SD
	Insulin (*μ*IU × h/ml)			Insulin (*μ*IU* *×* *h/ml per kcal)		
Obese	4869 ± 2784	3981 ± 2171	2349 ± 1004	23.3 ± 13.3	22.2 ± 12.1	3.2 ± 1.4
Normal-weight	3929 ± 1719	3046 ± 1406	2231 ± 509	18.8 ± 8.2	17.0 ± 7.8	3.0 ± 0.7

	Glucagon (ng* *×* *h/ml)			Glucagon (ng × h/ml per kcal)		
Obese	921 ± 356	926 ± 262	926 ± 262	4.4 ± 1.7	5.3 ± 2.1	1.2 ± 0.3
Normal-weight	1196 ± 514	1360 ± 662	1792 ± 1176	5.7 ± 2.5	7.6 ± 3.7	2.4 ± 1.6

## Data Availability

Database is stored by statistitian and available on reasonable request.
